# Effects of Apigenin Treatment on Random Skin Flap Survival in Rats

**DOI:** 10.3389/fphar.2021.625733

**Published:** 2021-02-24

**Authors:** Xinyi Ma, Yuting Lin, yingying Liu, Wenjie Li, Jibing He, Miaojie Fang, Dingsheng Lin

**Affiliations:** ^1^ Department of Hand and Plastic Surgery, The Second Affiliated Hospital and Yuying Children’s Hospital of Wenzhou Medical University, Wenzhou, China; ^2^ Second College of Clinical Medical, Wenzhou Medical University, Wenzhou, China; ^3^ First College of Clinical Medical, Wenzhou Medical University, Wenzhou, China

**Keywords:** apigenin, random skin flap, inflammation, oxidative stress, microangiogenesis

## Abstract

Random skin flaps are often used in plastic surgery, but the complications of marginal flap ischemia and necrosis often limit their wider clinical application. Apigenin (Api) is a flavonoid found in various fruits and vegetables. Api has been shown to promote angiogenesis, as well as reduce oxidative stress, membrane damage, and inflammation. In this study, we assessed the effects of Api treatment on random skin flap survival. Dorsal McFarlane skin flaps were transplanted into rats, which were randomly divided into three groups: control (normal saline), low-dose Api (20 mg/kg), and high-dose Api (50 mg/kg). Seven days after the surgery, the activity of superoxide dismutase (SOD) and the level of malondialdehyde (MDA) were measured. Histological analyses were performed to determine flap survival and tissue edema. H&E staining was performed to assess the histopathological changes in skin flaps, and the levels of microvascular density (MVD) were determined. Laser doppler flowmetry was used to assess microcirculation blood flow. Flap angiography was performed by injection of lead oxide/gelatin. The expression levels of vascular endothelial growth factor (VEGF), tumor necrosis factor-α (TNF-α), interleukin-6 (IL-6), and interlukin-1β (IL-lβ) were evaluated by immunohistochemistry. Rats in the high-dose Api group exhibited higher average flap survival area, microcirculatory flow, increased SOD activity, and higher VEGF expression levels compared with the other two groups. Furthermore, the levels of MDA and pro-inflammatory cytokines were significantly decreased in rats treated with high-dose Api. Our findings suggest the potential usefulness of Api in preventing skin flap tissue necrosis.

## Introduction

In contrast to axial pattern flaps, random skin flaps lack axial vascularization, due to anatomical differences in blood supply. Flap design and direction are not affected by the distribution of axial blood vessels; therefore, flaps are designed in a way to minimize tissue or organ dislocation during plastic surgery and cosmetic defect repairment. Since random skin flaps lack specific blood vessels, flap survival relies on blood supply from the pedicle muscle skin. Hence, low perfusion pressure in these tissues impacts flap survival, and marginal flap ischemia and necrosis, observed in 10–15% of cases (Dolan et al., 1995; Lille et al., 1999), limit their wide clinical application.

The development of interventions to prevent or treat skin flap necrosis remains an unmet clinical need. The length-to-width ratio of the flap should not exceed 2:1 because the lack of flap vascularization increases the risk of distal ischemia and necrosis. In the face and neck, the length-to-width ratio can be increased to 2.5:1, due to the good vascularization of these tissues. In random skin flaps, the scarfskin of the terminal necrotic area is characterized by marked inflammatory cell infiltration and coagulation necrosis (Ekin et al., 2019). Furthermore, inflammatory responses and ischemia reperfusion injury contribute to random skin flap necrosis. Ischemic flap tissues are characterized by extensive cell membrane damage and increased levels of arachidonic acid metabolites, promoting the recruitment of neutrophils and other inflammatory cells. The production of reactive oxygen species (ROS) and other inflammatory mediators by these immune cells aggravate ischemia and tissue damage (Kang et al., 2014). Therefore, the development of strategies to promote angiogenesis and improve local blood supply, as well as inhibit the production of inflammatory mediators and ROS, is crucial for improving the survival of random skin flaps.

Apigenin (Api) or 4′,5,7-trihydroxyflavone is a naturally occurring flavonoid, abundant in various vegetables, fruits, beans, and tea leaves, among which celery has the highest content. Api contains 4′ hydroxyl groups at positions 5 and 7, as well as a C2C3 double bond largely responsible for its unique physicochemical properties (Miean and Mohamed, 2001; Skerget et al., 2005). Although Api is not water-soluble, it can be dissolved in ethanol, dimethyl sulfoxide (DMSO), and low-concentration potassium hydroxide solution. Pure Api has a light yellow or yellow-green color. The phenol ring of Api can bind to different sugar groups, including glucose and glycoside ligands. Celery, an Api-rich plant, contains high levels of antioxidants, which have been shown to reduce oxidative stress and protect from numerous human diseases, including cancer (Nabavi et al., 2015), coronary heart disease (Hu et al., 2015), and aging. Api exerts anti-inflammatory effects by inhibiting immune cell production of tumor necrosis factor-α (TNF-α), interlukin-6 (IL-6), and interlukin-1β (IL-1β) production. Api promotes vascular regeneration after ischemia by enhancing the expression of vascular endothelial growth factor (VEGF) (Miean and Mohamed, 2001; Skerget et al., 2005). As a natural antioxidant, Api is also believed to lower blood pressure, prevent atherosclerosis, and suppress tumor growth (Telange et al., 2017). Compared with other flavonoids, Api has low toxicity and mutagenicity (Xu P. F. et al., 2017).

Due to its anti-inflammatory, antioxidant, and pro-angiogenic effects, Api treatment has been considered as a method to improve random skin flap survival (Bachle et al., 2011; Krag et al., 2017; Pang et al., 2018; Ren et al., 2018). In this study, we established a rat model of random skin flap, and assessed the effects of Api on flap survival.

## Materials and Methods

### Animals and Reagents

Ethical approval for this study was provided by the Laboratory Animal Ethics Committee of Wenzhou Medical University, Wenzhou, China (Chairperson, Prof. Shengwei Jin) on February 28, 2018 (no. WYDW2017-0509). Pathogen-free Sprague–Dawley male rats (200–250 g, 2–3 months old) were obtained from the Wenzhou Medical University Laboratory Animal Center. Api (purity ≥98%) was purchased from Solarbio life sciences (Beijing, China). Api was dissolved in 4% (v/v) DMSO, and corn oil was added to achieve a concentration of 4 mg/ml.

### Establishment of a Rat Flap Model

**FIGURE 1:**
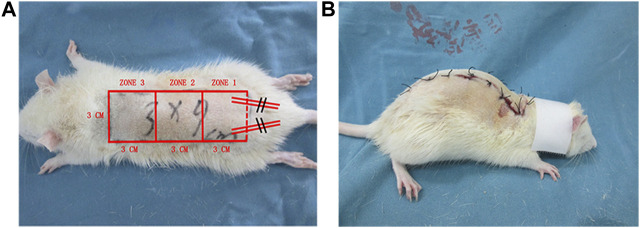
**(A)** A modified McFarlane flap was established on the dorsum of each rat. **(B)** A patent neck cover was used to prevent the rat from chewing the flap.

Rats (n = 60) were divided into three groups: high-dose (50 mg/kg) Api, low-dose (20 mg/kg) Api, and control (n = 20 animals per group). Rats were not fed the night before the experiment. The next day, rats were anesthetized by intraperitoneal injection of pentobarbital sodium saline solution (40 mg/kg). After back hair removal, the modified McFarlane (3 × 9 cm) flap was applied to the middle back of each rat (Figure 1A). The pedicle artery perforator flaps were sutured with 4-0 nylon sutures. To prevent shock, we intraperitoneally injected 50 ml/kg of normal saline. For postoperative observation, the flaps were divided into three types: proximal area (zone 1), middle area (zone 2), and distal area (zone 3). After surgery, rats were single-caged (25°C, 40–60% humidity) and given ad libitum access to water and food. Rats in the high-dose Api and low-dose Api groups were intragastrically administered 20 and 50 mg/kg Api, respectively, per day; rats in the control group were administered corn oil and DMSO. The operation was performed daily at 3 pm for seven consecutive days. All procedures were performed by the same person to minimize experimental errors.

### Macroscopic Flap Survival Assessment

Flap survival was macroscopically examined at days 1, 3, 5, and 7; changes in color, texture, tissue elasticity, hair growth, and tissue necrosis were recorded. Skin flap necrosis was defined as blackened color, stiff texture, shrunken tissue, reduced elasticity, and absence of bleeding after tissue puncture. The flap survival area was assessed 7 days after the operation by measuring the surviving area and total area of each flap using weighing paper and the weights of the flap using an electronic scale. Flap survival quantification was achieved using the following formula: flap survival area (%) = paperweight of the flap survival area/paperweight of the total flap surface area × 100.

### Histopathological Examination

Rats were sacrificed 7 days after the operation by anesthetic overdose. Tissue samples (1 × 1 cm) were obtained from the proximal, middle, and terminal skin flaps (zones 1, 2, and 3, respectively), fixed in 4% paraformaldehyde for 24 h, and paraffin-embedded. Tissue sections were prepared, followed by dewaxing, dehydration, staining, and microscopic examination. The degrees of tissue edema, necrosis, and neutrophil infiltration were evaluated under a light microscope (magnification, ×100). Microvascular density (MVD) was used to assess angiogenic aggregation. Five optical fields were randomly selected for each tissue section, and the microvessel number in the cross-section was counted; the average number of microvessels per square millimeter area was calculated and the microvessel density per unit area was used to evaluate MVD.

### Laser Doppler Flowmetry

Seven days after the operation, rats were anesthetized and restrained. The blood flow in each area of the flap (proximal area, middle area, and distal area) was measured using a laser Doppler imaging system. Microcirculatory blood flow in the local tissues was expressed as perfusion units (PU).

### Gelatin/Lead Oxide Angiography

Rats were sacrificed 7 days after the operation by anesthetic overdose. Pre-warmed (37°C) isotonic saline was injected into the carotid artery to clean blood vessels, and the blood was drained through the jugular vein; body temperature was monitored throughout this process. Subsequently, gelatin/lead oxide solution (100 ml/kg) was injected into the carotid artery; perfusion was continued until the solution reached the limbs, ears, and corneas. The dorsal flap and cutis were dissected and subjected to X-ray angiography.

### Immunohistochemical Staining

Paraffin-embedded sections were stained using the Elivison two-step method. Sections were incubated with normal goat serum blocking solution and left to stand at room temperature (20–25°C) for 25 min. Tissue sections were then incubated with a mouse anti-rat VEGF primary antibody (1:100 in 50 μl) overnight at 4°C. Subsequently, sections were incubated at 37°C for 45 min, washed with phosphate-buffered saline (PBS), and incubated with a goat anti-mouse secondary antibody (1:50 in 50 μl) for 1 h at 37°C. After washing with PBS, tissue sections were incubated with diaminobenzidine for 10 min. Sections were observed under low-power and high-power light microscopes. Five optical fields per section were imaged and used for quantification of VEGF expression. The basic principle of field selection in the immunohistochemical analysis was to randomly select five fields that did not overlap under a 400× light microscope. Similar methods were used to detect the expression level of TNF-α,IL-6 and IL-1β.

### TNF-α and IL-6 Serum Levels

Blood was centrifuged at 5,000 × g for 15 min, serum was collected, and serum TNF-α and IL-6 levels were measured using an ELISA kit according to the manufacturer’s instructions. Optical absorbance was measured at 450 nm.

### Superoxide Dismutase (SOD) Activity and Malondialdehyde (MDA) Content Measurements

Ischemia reperfusion injury typically occurs within 24–48 h of flap transplantation. The rats were sacrificed by anesthetic overdose 7 days after the surgery. Tissue samples (0.5 × 0.5 cm) were collected from the middle area (zone 2), where partial flap necrosis was observed. Samples were homogenized, weighed, and diluted in an ice bath to 10% of their initial concentration. The MDA level and SOD activity were measured according to the manufacturer’s instructions.

### Statistical Analysis

Statistical analyses were performed using SPSS version 19 (Chicago, IL, United States). Data were expressed as mean ± standard error of the mean (SEM). The mean values of different groups were compared using the Student’s *t*-test. *p*-values < 0.05 were considered statistically significant.

## Results

### Effects of Api on Flap Survival and Features

**FIGURE 2:**
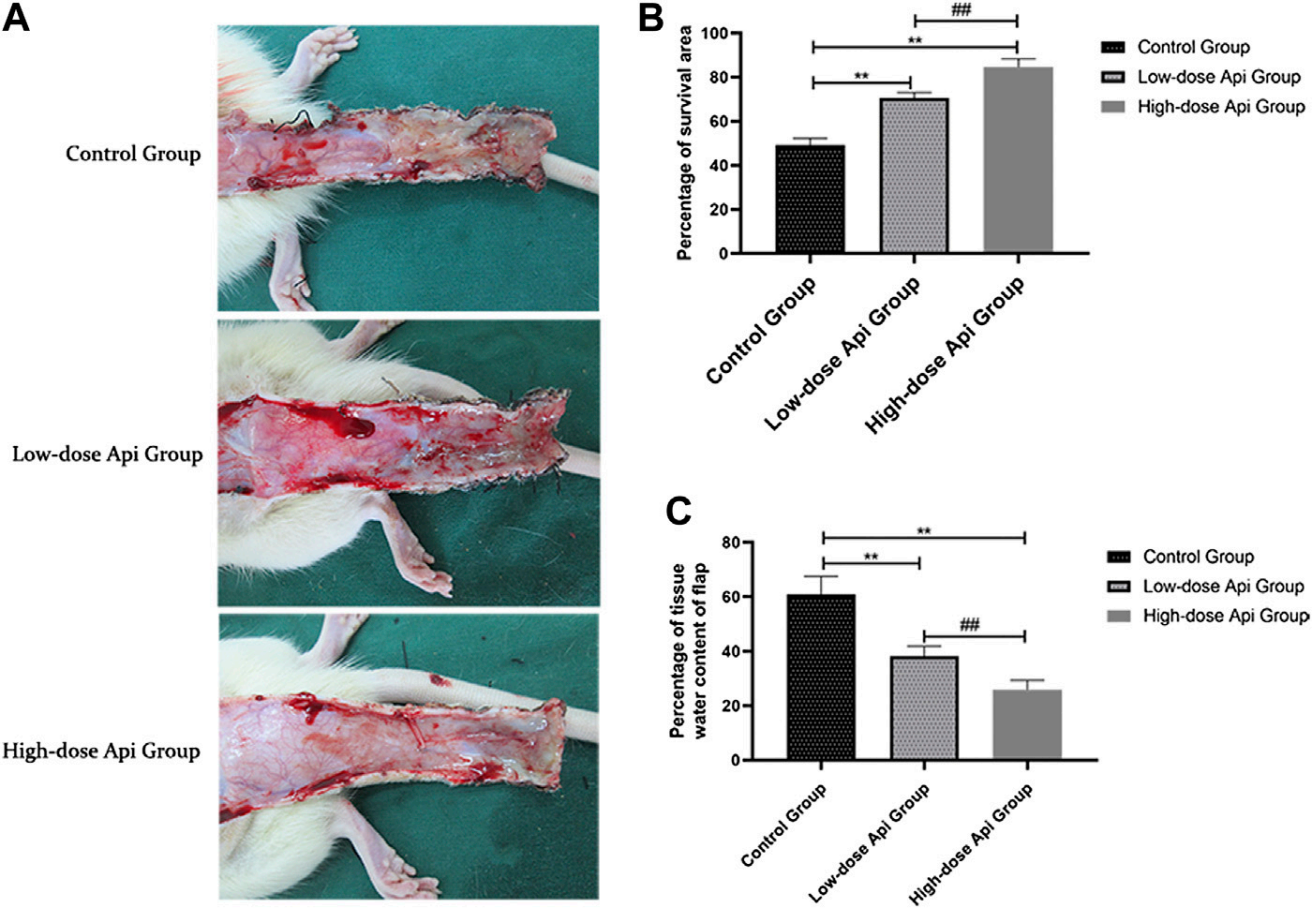
**(A)** Digital photographs of dorsal skin flaps in rats from the three groups. Macroscopic comparison of the overall appearance of the surviving and necrotic areas of the flap. **(B)** Percentage of survival area on day 7. Api increased the survival rate of skin flaps. **(C)** Percentage of tissue water content on day 7. Api reduced the degree of tissue edema in skin flaps.**p < 0.01 vs. control group. ^##^ *p* < 0.01 vs. Low-dose Api group.

On postoperative day 1, all animals presented some degree of swelling near the incision edges of zone 3, accompanied by dark brown patches. However, there was no profound tissue swelling in zones 1 and 2. Seven days after the operation, we observed a demarcation line between the healthy area of the flap and the necrotic tissue. The necrotic tissue, especially in the distal flap, was dark and hard, with reduced tissue elasticity and granulation. Macroscopic examination revealed that the extent of necrosis was greater in the control group compared to that in Api-treated rats (Figure 2A). The flap survival rate was markedly reduced in the control group (49.18 ± 2.83%) compared to that in the low-dose Api and high-dose Api groups (70.66 ± 2.18 and 84.51 ± 3.85%, respectively; Figure 2B). The degree of tissue edema was evaluated by measuring the tissue water content. The percent tissue water in the high-dose Api, low-dose Api, and control groups were 25.86 ± 3.49, 38.23 ± 3.61, and 60.88% ± 6.59%, respectively (Figure 2C). The differences in the extent of tissue edema among the groups were statistically significant (*p* < 0.01).

### Api Prevents Histopathological Skin Flap Damage

**FIGURE 3:**
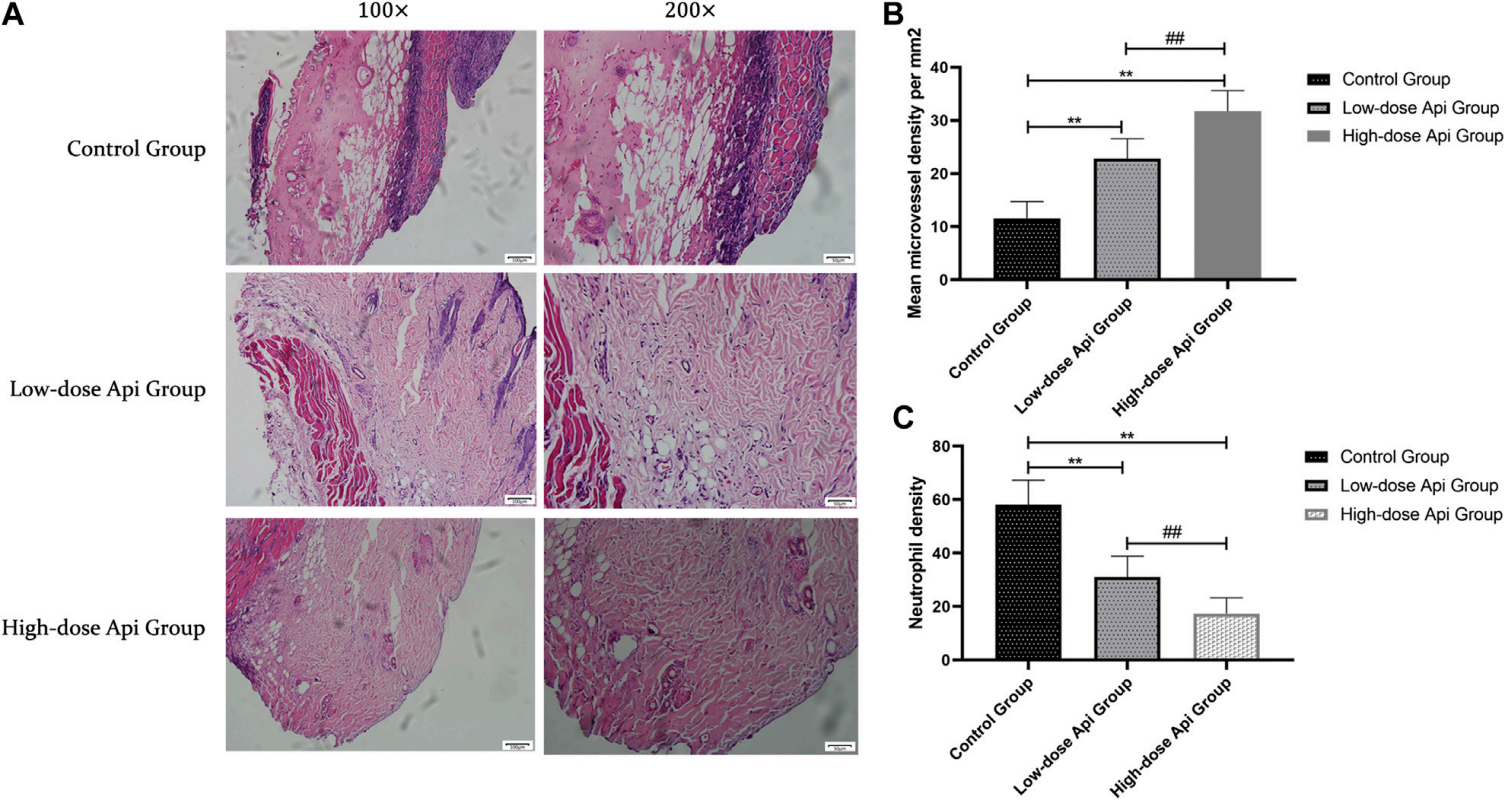
**(A)** Histopathological features of the flaps were assessed using hematoxylin and eosin (H&E) staining on day 7 after the operation; representative images acquired with a light microscope are shown (magnification,×100, ×200). Api reduced histopathological damage. **(B)** The microvascular density in zone 2 of the dorsal flap on day 7 after the operation. Api increased microvessel density. **(C)** Neutrophil density on day 7 after the operation. Api reduced neutrophil density.**p < 0.01 vs. control group. ^##^ *p* < 0.01 vs. Low-dose Api group.

In contrast with the flaps of Api-treated animals, the flaps of the control rats presented profound structural and histological abnormalities, including excessive inflammatory cell infiltration, reduced neovascularization, reduced fibroblast proliferation, granulation tissue thinning, and tissue edema (Figure 3A). Furthermore, the MVDs of zone II in the high-dose Api group (31.75 ± 3.85/mm^2^) and low-dose Api group (22.81 ± 3.75/mm^2^) were significantly higher than that in the control group (11.58 ± 3.12/mm^2^; *p* < 0.01; Figure 3B). Moreover, the neutrophil density in the low-dose Api group (31.05 ± 7.79/mm^2^) and high-dose Api group (17.37 ± 5.83/mm^2^) was significantly lower than that in the control group (58.07 ± 9.26/mm^2^; *p* < 0.01; Figure 3C).

### Api Improves Blood Flow in Zone 2

**FIGURE 4:**
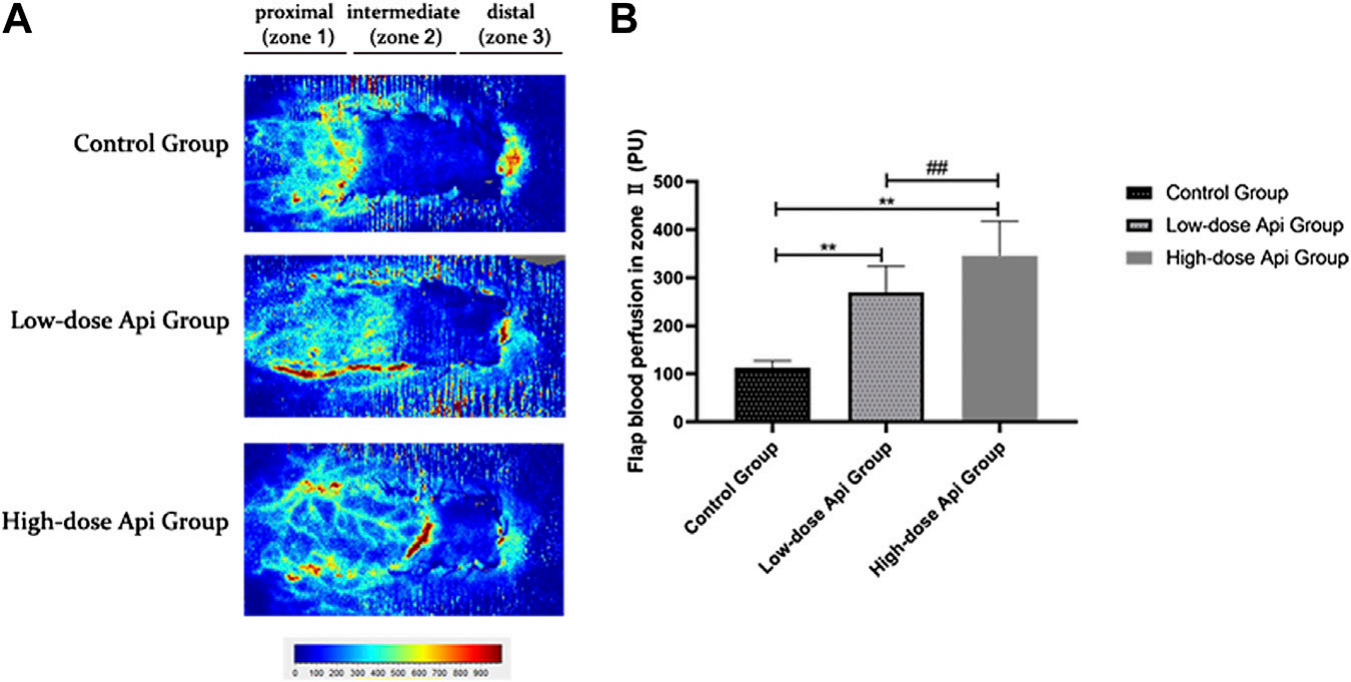
**(A)** Laser Doppler flowmetry angiography showing blood perfusion on day 7 in the different groups. **(B)** Quantification of blood perfusion in zone 2 of the flap. Api increased blood flow. **p < 0.01 vs. control group. ^##^ *p* < 0.01 vs. Low-dose Api group.

LDF angiography revealed that flap blood perfusion in zone 2 was significantly higher in the low-dose Api group (269.72 ± 54.58 PU) and high-dose Api group (345.65 ± 71.87 PU) compared with that in the control group (112.53 ± 14.88 PU; *p* < 0.01; Figure 4).

### Api Promotes Skin Flap Neovascularization

**FIGURE 5:**
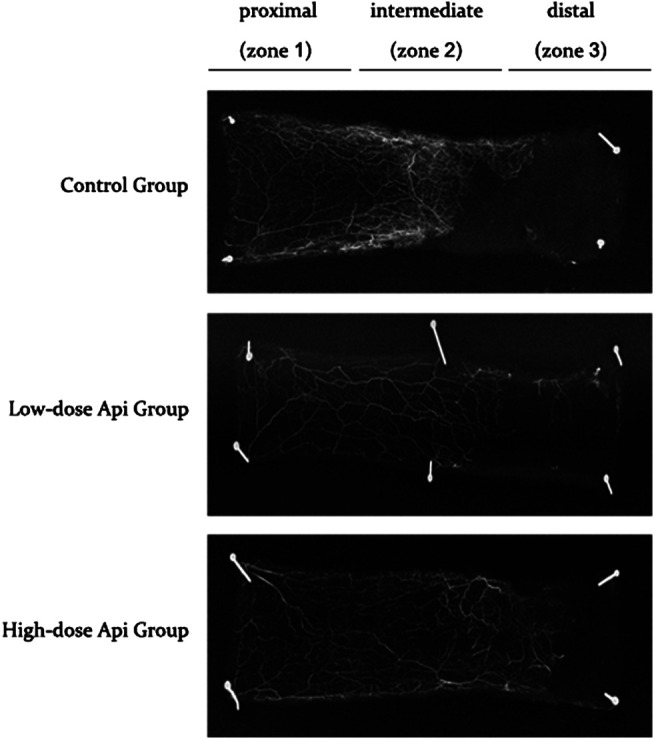
X-ray angiography of the flaps in the control, low-dose Api, and high-dose Api groups on day 7 after the operation. Api improved flap angiographic features.

Gelatine/lead oxide angiography 7 days after the operation indicated that neovascularization in the low-dose and high-dose Api groups was significantly higher than in the control group (Figure 5).

### Effects of Api on VEGF, IL-6, IL-1β, and TNF-α Levels

**FIGURE 6:**
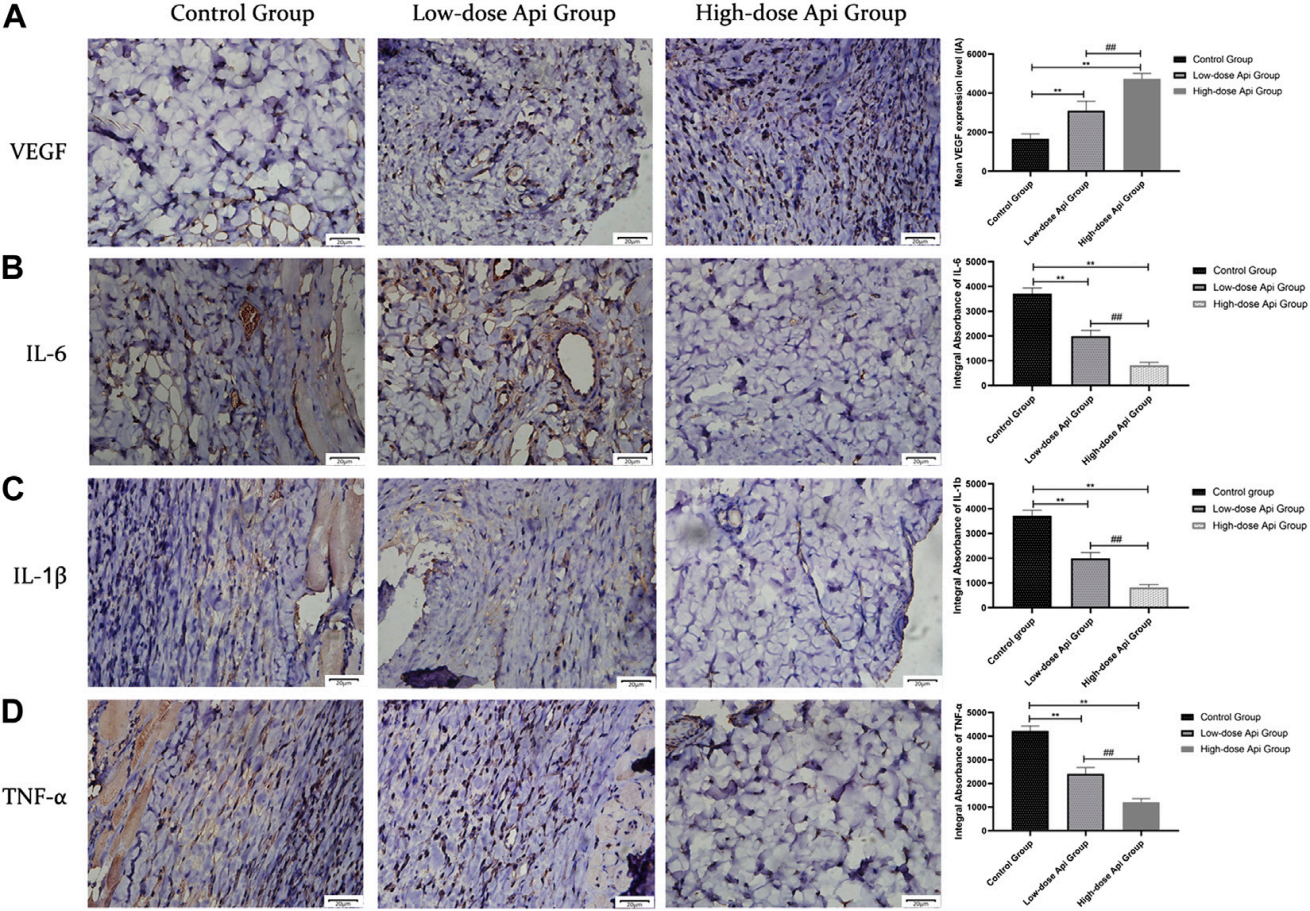
Representative images after immunohistochemical staining for different pro-inflammatory cytokines 7 days after the operation (magnification, ×400). Expression levels of VEGF, TNF-α, IL-1β, and IL-6 in zone 2 of dorsal flaps. Api promoted the expression of VEGF and inhibited the expression of IL-1β, IL-6, and TNF-α. **p < 0.01 vs. control group. ^##^ *p* < 0.01 vs. Low-dose Api group.

Immunohistochemical observations revealed that Api enhanced VEGF expression and downregulated the expression of pro-inflammatory cytokines in a dose-dependent manner. The levels of VEGF in the high-dose Api, low-dose Api, and control groups were 4,737.67 ± 267.01 IA, 3,090.00 ± 493.32 IA, and 1,665.83 ± 248.91 IA, respectively (*p* < 0.01). The levels of IL-6 in the high-dose Api, low-dose Api, and control groups were 819.83 ± 115.31 IA, 1,996.33 ± 230.22 IA, and 3,710.17 ± 225.38 IA, respectively (*p* < 0.01). Similarly, IL-1β levels in the high-dose Api, low-dose Api, and control groups were 721.67 ± 105.91 IA, 1,293.83 ± 239.67 IA, and 2,253.33 ± 373.67 IA, respectively (*p* < 0.01). The levels of TNF-α in the high-dose Api, low-dose Api, and control groups were 1,196.33 ± 159.29 IA, 2,402.5 ± 271.01 IA, and 4,223.00 ± 206.92 IA, respectively (*p* < 0.01; Figure 6).

### Effects of Api on Pro-Inflammatory Cytokine Production

**FIGURE 7:**
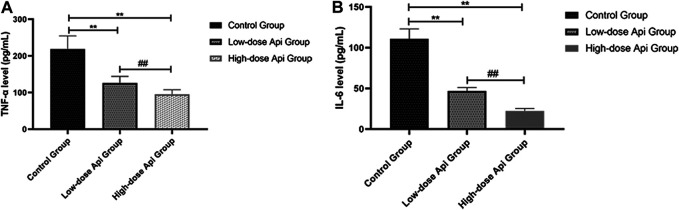
Serum levels of TNF-α **(A)** and IL-6 **(B)** 7 days after the operation as determined by ELISA. Api inhibited the expression of TNF-α and IL-6.**p < 0.01 vs. control group. ^##^ *p* < 0.01 vs. Low-dose Api group.

The serum levels of pro-inflammatory cytokines were determined by ELISA. We found that the levels of TNF-α were significantly lower in the high-dose Api group (95.62 ± 12.11 pg/ml) and low-dose Api group (125.83 ± 18.65 pg/ml) compared with those in the control group (218.36 ± 36.21 pg/ml; *p* < 0.01; Figure 7A). Furthermore, serum IL-6 levels were profoundly lower in the high-dose Api group (22.25 ± 3.11 pg/ml) and low-dose Api group (46.89 ± 4.12 pg/ml) compared with those in the control group (110.93 ± 12.08 pg/ml; *p* < 0.01; Figure 7B).

### Effects of Api on SOD Activity and the MDA Level

**FIGURE 8:**
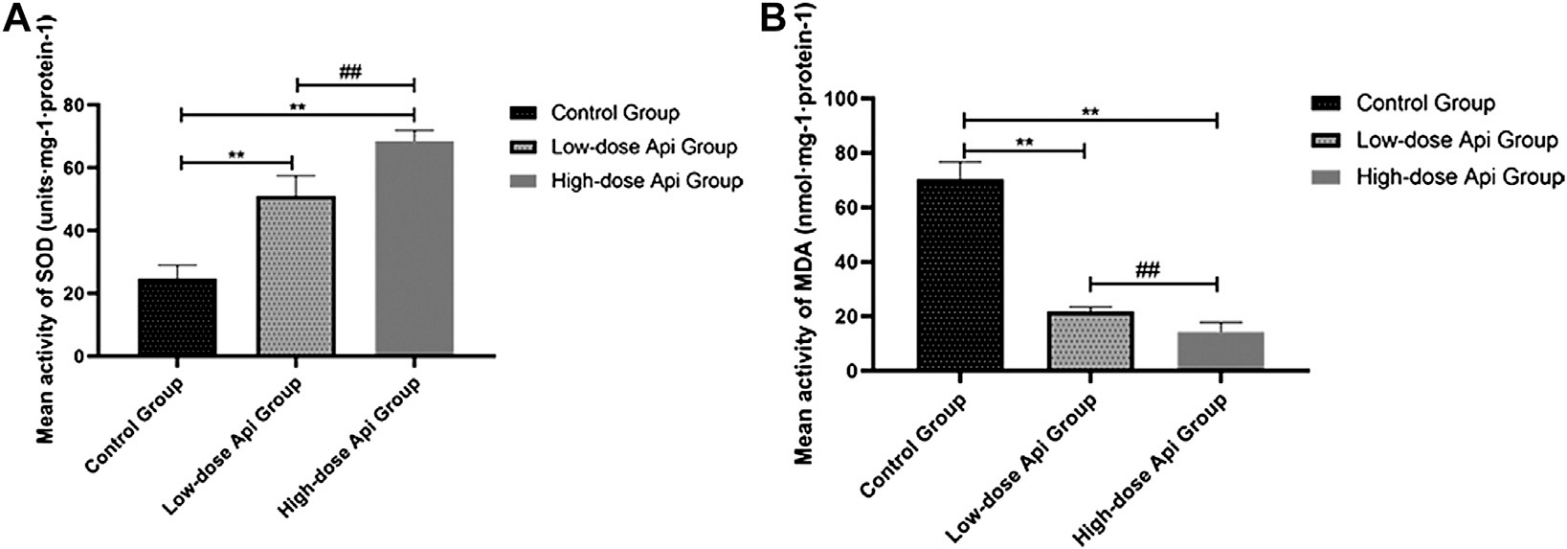
**(A)** Mean SOD activity on day 7. **(B)** Mean MDA content on day 7. Api enhanced SOD activity and inhibited the expression of MDA.**p < 0.01 vs. control group. ^##^ *p* < 0.01 vs. Low-dose Api group.

Api treatment enhanced SOD activity and reduced the MDA level in a dose-dependent manner. Particularly, SOD activity in the low-dose Api group (51.12 ± 6.36 units·mg-1·protein-1) and high-dose Api group (68.52 ± 3.42 units·mg-1·protein-1) were significantly higher compared with that in the control group (24.81 ± 4.23 units·mg-1·protein-1; *p* < 0.01; Figure 8A). Conversely, MDA levels in the low-dose Api group (21.89 ± 1.60 units·mg-1·protein-1) and high-dose Api group (14.29 ± 3.53 units·mg-1·protein-1) were significantly lower compared with those in the control group (70.49 ± 6.25 units·mg-1·protein-1; *p* < 0.01; Figure 8B).

## Discussion

Api is a flavonoid found at higher levels in celery. Several lines of evidence have shown that it promotes tissue healing and angiogenesis, and prevents venous thrombosis. Additionally, Api can reduce oxidative stress and inflammation and has an excellent safety profile in humans (Ren et al., 2018; Salehi et al., 2019; Zhou et al., 2019). A previous study showed that Api (20 mg/kg/day) administration significantly attenuated early brain injury, which includes brain edema, blood–brain barrier disruption, neurological deficiency, and cell apoptosis, after subarachnoid hemorrhage in rats by suppressing the expression of toll-like receptor 4 (TLR4), nuclear factor-κB (NF-κB), and their downstream pro-inflammatory cytokines in the cortex and by upregulating the expression of tight junction proteins of the blood–brain barrier (Zhang et al., 2015). In addition, a diabetes study showed that Api (20 mg/kg/day) improves renal dysfunction and oxidative stress (Malik et al., 2017). It also prevented mitogen-activated protein kinase activation, which inhibited inflammation (reduced TNF-α, IL-6, and NF-κB expression) and apoptosis (increased expression of Bcl-2 and decreased expression of Bax and caspase-3). Api (50 mg/kg/day) significantly improved paraquat-induced acute lung injury in mice (Luan et al., 2016). These findings indicate that Api administration decreases biochemical parameters of inflammation and oxidative stress, and improves oxygenation and lung edema, in a dose-dependent manner. All of these effects are likely to improve the survival of ischemic flaps; thus, we chose 20 and 50 mg/kg/day as therapeutic Api doses.

In this study, we showed that intragastrical administration of Api in rats induced the expression of VEGF, promoting angiogenesis. Additionally, it reduced oxidative stress and tissue inflammation, as well as inhibited ischemia reperfusion injury in skin flaps, reducing tissue edema, and improving flap survival. Our team has been exploring methods to intervene in the necrosis of ischemic flaps and has found that azadirachtin A (He et al., 2020), dexmedetomidine (Fang et al., 2020), and nobiletin (Jiang et al., 2020) have positive effects on flap survival. However, Api has better anti-inflammatory effects, reducing the release of inflammatory cytokines and inhibiting the chemotaxis and adhesion of neutrophils. In addition, Api, as a natural flavonoid (Salehi et al., 2019) extracted from fruits and vegetables, has a wide range of sources and can be obtained easily through the daily diet. It is less expensive than most synthetic drugs and has fewer side effects, which are major advantages for Api as a potential new drug to promote flap survival.

The excision of a large tissue area from the donor is vital for sufficient blood supply in the skin flap and subcutaneous adipose tissue (Wu et al., 2019), which, after transplantation, is initially supplied by nutrients from the donor tissues. Tissue necrosis is the most common complication after skin flap surgery. In contrast to axial pattern flaps, random skin flaps lack axial vascularization and are, therefore, dependent on existing vessels in the surrounding tissues (Cai et al., 2015). However, maintaining a low length-to-width ratio in random skin flaps is crucial for flap survival, as the distal end of the flap may suffer from nutrient deprivation, leading to necrosis (Memarzadeh et al., 2016). Flap survival primarily depends on blood perfusion pressure. In random skin flaps, pedicle muscle vessels are the primary source of blood and nutrients. Therefore, the survival of ultra-long random skin flaps requires constant blood flow at the flap ends, which allows for the formation of new capillaries in the flap (Wang et al., 2011; Ciudad et al., 2017). Although longer operation times can increase flap blood supply and improve flap survival and quality, it can also increase the risk of tissue injury. Therefore, delays in the transplantation process can greatly impact flap survival (Murphy et al., 1985; Haykal et al., 2018; Arslan and Demiroz, 2019; Aydin et al., 2019).

Inflammatory responses also play decisive roles in the survival of random skin flaps. Necrotic areas at the distal flap are characterized by profound coagulative necrosis and inflammatory cell infiltration. Additionally, the extent of tissue necrosis has been associated with the degree of inflammatory responses (Perry et al., 2018). Hence, preventing immune responses and inflammation can improve flap survival. The mechanisms underlying tissue necrosis, inflammatory responses, and ischemia reperfusion injury form a complex network that impairs flap survival (Ma et al., 2018). Among these mechanisms, those involved in ischemia reperfusion injury are believed to act upstream in this network. Hypoxic and ischemic flap tissues often exhibit extensive cell membrane damage. During reperfusion, membrane damage and the subsequent rise in the levels of arachidonic acid are further increased (Zachary et al., 1979), promoting neutrophil recruitment and aggravating inflammation. Flap-infiltrating neutrophils produce high levels of ROS, exacerbating tissue damage and inflammation.

Mounting evidence suggests that Api has remarkable anti-inflammatory effects. Du H et al. showed that the Api-rich plant celery strongly suppressed inflammation in rat models, mitigating acute myocardial infarction (Du et al., 2015). Moreover, Li K et al. demonstrated that celery inhibited Toll-like receptor 4 (TLR4) signaling, protecting against acute lung injury after lipopolysaccharide treatment (Li et al., 2018). Pang Q et al. showed that celery alleviated ischemia reperfusion injury in the brain by activating the caveolin-1/VEGF axis (Pang et al., 2018)10. In this study, we found that Api treatment reduced the levels of numerous pro-inflammatory cytokines, including TNF-α, IL-6, and IL-1β. This finding suggests that Api may alleviate inflammation by suppressing the production of pro-inflammatory cytokines and other inflammatory mediators (Charalabopoulos et al., 2019).

Ischemia reperfusion injury is another factor promoting ROS production and oxidative stress and is considered to be the leading cause of skin flap necrosis (Georgopoulos et al., 2012). As an antioxidant, SOD scavenges superoxide anion radicals, thereby maintaining free radical homeostasis during ischemia reperfusion injury (Xu Y. et al., 2017). By contrast, MDA induces membrane damage. Previous studies have shown that Api alleviated cardiac ischemia reperfusion injury (Feng et al., 2018). In this study, we found that Api significantly increased SOD activity and reduced MDA content, suggesting that Api can prevent ischemia reperfusion injury by reducing oxidative stress (Huang et al., 2019).

We also found that Api upregulated VEGF expression. VEGF levels have been associated with vascular regeneration in skin flaps. Early during angiogenesis, VEGF promotes vascular endothelial cell proliferation and differentiation (Gao et al., 2015; Cochran et al., 2016). Previous studies have shown that VEGF reduced skin flap necrosis, prevented flap infection, promoted wound healing, and improved flap survival (Alkharsah 2018; An et al., 2018; Melincovici et al., 2018). In this study, hematoxylin and eosin staining and gelatine/lead oxide angiography results indicated that Api treatment enhanced neovascularization. The density of capillaries in the high-dose Api group was higher than in the low-dose Api group, suggesting a dose-dependent effect. Improved blood flow and nutrient supply can reduce inflammation and improve random skin flap survival.

Compared with other flavonoids, such as quercetin (Mekjaruskul and Sripanidkulchai, 2015; Carullo et al., 2017) and kaempferia brass (Mekjaruskul and Sripanidkulchai, 2015), Api has an improved safety profile. Owing to its anti-inflammatory, antioxidant, and pro-angiogenic effects, Api has various applications in medicine and the food industry (Brad and Zhang, 2018). However, low water-solubility and intestinal absorption contribute to the low bioavailability of orally administered Api, limiting its wide application. Moreover, future studies are required to establish the doses of Api that provide maximum clinical benefits.

We conclude that Api improves flap survival, but the underlying mechanism remains to be elucidated fully. Based on existing research, we believe that this mechanism may be related to the TLR4 or caveolin-1/VEGF pathway, which we will consider in future research.

## Conclusion

Given its potent antioxidative, angiogenic, and anti-inflammatory capabilities, Api has potential applications for the treatment of ischemic flap tissue in rats. Further studies are necessary to determine the optimal dose and evaluate the safety of Api as a drug, as well as to yield a more detailed understanding of the drug’s mechanisms of action. This study was an experimental investigation performed exclusively in rats; whether Api can be applied to the recovery and reconstruction of human skin flap transplantation requires further investigation.

## Data Availability

The original contributions presented in the study are included in the article/Supplementary Material, further inquiries can be directed to the corresponding author.
